# Viscoelastic Mechanical Responses of HMAP under Moving Load

**DOI:** 10.3390/ma11122490

**Published:** 2018-12-07

**Authors:** Yazhen Sun, Bincheng Gu, Lin Gao, Linjiang Li, Rui Guo, Qingqing Yue, Jinchang Wang

**Affiliations:** 1 School of Transportation Engineering, Shenyang Jianzhu University, Shenyang 110168, China; gbc0207@stu.sjzu.edu.cn (B.G.); 1414304706@stu.sjzu.edu.cn (L.L.); gr329@stu.sjzu.edu.cn (R.G.); yqq@stu.sjzu.edu.cn (Q.Y.); 2 College of Architecture Engineering, Chongqing University of Arts and Sciences, Chongqing 402160, China; gaolin32@163.com; 3 Institute of Transportation Engineering, Zhejiang University, Hangzhou 3100580, China

**Keywords:** high-modulus asphalt mixture (HMAM), dynamic tests, viscoelasticity, dynamic responses, resistance to deformations, tensile strains, tensile stresses, sensitivity analysis

## Abstract

In order to represent the mechanical response laws of high-modulus asphalt pavement (HMAP) faithfully and objectively, the viscoelasticity of high-modulus asphalt mixture (HMAM) was considered, and the viscoelastic mechanical responses were calculated systematically based on moving load by numerical simulations. The performances of the HMAP in resistance to the deformation and the cracking at the bottom layer were compared with the ordinary asphalt pavement. Firstly, Lubao and Honeywell 7686 (H7686) were selected as the high modulus modifiers. The laboratory investigations of Asphalt mix-70 penetration, Asphalt mix-SBS (styrene-butadiene-styrene), HMAM-Lubao and HMAM-H7686 were carried out by dynamic modulus tests and wheel tracking tests. The conventional performances related to the purpose of using the HMAM were indicated. The master curves of the storage moduli were obtained and the viscoelastic parameters were fitted based on viscoelastic theories. Secondly, 3D pavement models based on moving loads for the viscoelastic structures were built using the non-linear finite element software ABAQUS. The wheel path was discretized in time and space to apply the Haversine wave load, and then the mechanical responses of four kinds of asphalt pavement were calculated. Finally, the sensitivity analysis was carried out. The results showed that the addition of the high modulus modifiers can improve the resistance to high-temperature rutting of the pavements. Except for the tensile strain and stress at the bottom of the underlayer, other responses decreased with the increases of the dynamic moduli and the change laws of the tensile strain and stress were affected by the range of the dynamic modulus. The tensile stress at the bottom of the asphalt layer would be too large if the modulus of the layer were too large, and a larger tensile strain would result. Therefore, the range of the modulus must be restricted to avoid the cracking due to excessive tension when using the HMAM. The resistance of the HMAP to deformation was better and the HMAP was less sensitive to load changes and could better withstand the adverse effects inflicted by heavy loads.

## 1. Introduction

In roads with heavy traffic, the proportion of the damaged pavement is increasing [1,2,3,4]. Findings have demonstrated that increasing the modulus of the asphalt mixture was an effective way to resist damages and extend the service life of the pavements [5,6,7]. The-high modulus asphalt mixture (HMAM), first developed in France, a kind of hot asphalt mixture with a dynamic modulus (15 °C, 10 Hz) greater than 14,000 MPa, is being taking seriously by researchers. In China, the material was mainly made by directly adding high-modulus modifier into the aggregates, and the modulus of asphalt mixtures could be significantly increased and the resistance to the deformations improved [8]. The asphalt pavement is actually a typical viscoelastic structure and bears moving loads. However, the pavement structure models were built based on static loads and elastic layer systems by most researchers for numerical simulations, and the inertial forces and time dependency were not taken into account, which could not reflect the actual state of the pavement [9]. How to represent the dynamic properties of the pavement, the resistances to deformations and the cracking of the asphalt layer bottom when the HMAM was used for the layer are questions that need to be answered in the promotion of the high-modulus asphalt pavement (HMAP) in China. So it is imperative to apply moving wheel loads to the pavement based on the viscoelastic properties.

Until now, researchers have carried out many studies on the mechanical properties of the HMAP. The finite element method was used to compare the mechanical responses of high modulus and ordinary asphalt pavements under different axle loads, suggesting that some mechanical responses such as the compressive strains can be effectively reduced by the HMAM [10,11,12]. The dynamic modulus was directly applied to analyze the shearing stress and shearing strain of the HMAP, and the conclusion was drawn that the strain level of the pavement could be significantly reduced by increasing the modulus [13,14,15]. A full-scale test of the asphalt pavement under repeated loads was carried out to verify this [16,17]. The creep test results were fitted to the viscoelastic parameters based on the Burgers model and the static cyclic loads were applied to simulate the rutting formation, with the results showing that the rutting deformation of the HMAP was significantly less than that of ordinary asphalt pavement when the HMAM was used as the middle surface layer [18,19,20]. The dynamic moduli were calculated using the LEDFAA program; it was found that the HMAP could also slow down rutting formation [21]. It can be seen that the current research on the HMAP is mainly focused on its resistance to rutting and shearing stress, and other internal responses such as the tensile strain, need to be systematically analyzed. Due to the restrictions of the experimental conditions, in most studies, the asphalt pavement was assumed to be an elastic layered system, which did not reflect the viscoelasticities of the asphalt mixtures. In a dynamic analysis of the pavement, an important factor needs to be considered: the dependency of the material properties on the loading frequency [22]. The time-domain Prony series expression of the relaxation modulus can accurately represent the long-term complex viscoelastic behaviors of the asphalt mixtures. It is important to perform the dynamic analysis of flexible pavements subjected to traffic load. The study conducted by Al-Qadi [23] reported that the maximum differences between responses obtained by quasi-static and dynamic analyses were 39%, 25% and 10% for the tensile strain at the bottom of the HMAP, the compressive stress at the top of the subgrade and the longitudinal strain, respectively. The static cyclic load could not reflect the dynamic states of the road pavements. In view of this, in this paper, the viscoelastic parameters of the HMAM need to be obtained through a dynamic modulus test, and the viscoelastic pavement model under moving load should be built to systematically study the mechanical response laws of the HMAP.

The viscoelastic mechanical responses of ordinary asphalt pavements subjected to moving loads have been studied by scholars in recent years. Such responses as the compressive strain of the subgrade at different speeds were analyzed, demonstrating that the responses increased significantly at a low vehicle speed [24,25]. The viscoelastic mechanical properties of two typical thin and thick pavements sections at different speeds were studied and the results showed that the shearing stress has a certain influence on the tensile strain at the bottom of the asphalt layer [26,27]. Based on the measured structural parameters and vehicle characteristics, a 3D viscoelastic pavement model was built using the finite element software, ABAQUS, and the mechanical responses under different loads at different speeds were analyzed. Then, a comparison was made between the measured and calculated mechanical responses, demonstrating that the responses could be reasonably simulated by the model, the shearing stress and the shearing strain tended to be concentrated in the middle surface layer, and the viscoelastic properties led to the asymmetrical mechanical response curves [28,29,30]. The responses under moving loads based on the Laplace transform were solved by the Boltzmann superposition principle. The results were compared with those by numerical calculations to verify the feasibility of the simulation based on viscoelasticity [31]. It can be seen that the time-domain Prony series expression of the relaxation modulus used in this study can accurately represent the long-term complex viscoelastic behaviors of the asphalt mixtures. The previous analyses were carried out using ordinary asphalt mixtures, and there was a lack of objective and systematic analysis of the HMAP, and how to represent the mechanical responses of the HMAP and the characteristics of the stresses were not discussed. In addition, the problem of the changing characteristics of the stress of the HMAP compared with other pavements urgently needs to be solved in the promotion of the HMAP in China.

Based on the previous studies, the aim of this paper was to apply real viscoelastic parameters and moving loads to the HMAP, and 3D viscoelastic pavement models based on moving loads were built. More objective and systematic studies on the mechanical responses were conducted and compared with common pavement structures and the characteristics of the HMAP were analyzed. The following studies were conducted: laboratory investigations of four kinds of asphalt mixtures were carried out by wheel tracking tests and dynamic modulus tests. The resistance to high-temperature rutting of HMAM were indicated. The viscoelastic parameters were fitted based on the viscoelastic theories. The 3D viscoelastic dynamic models were built using ABAQUS. The mechanical response laws such as the vertical deformations and the longitudinal tensile strains under standard and heavy loads were studied. The characteristics of the HMAM and the sensitivities of various pavement structures to the level of the loads were analyzed in an objective way to provide the theoretical basis for improving the HMAP.

## 2. Dynamic Modulus Testing

### 2.1. Materials

The 70-Penetration asphalt and the styrene-butadiene-styrene (SBS) asphalt (in accordance with the Chinese standard of the Technical Specifications for Construction of Highway Asphalt Pavements (JTG F40—2004)), referred to as “asphalt binder”, were mixed with limestone, the aggregates to form materials used in asphalt pavements, and the properties were listed in Table 1 and Table 2. The two kinds of high modulus modifiers (Lubao and H7686) were directly added into the aggregates. The modifiers were blended first with hot aggregates and then mixed with hot asphalt and mineral filler to ensure uniform dispersion of the mixture.

**Figure 1:**
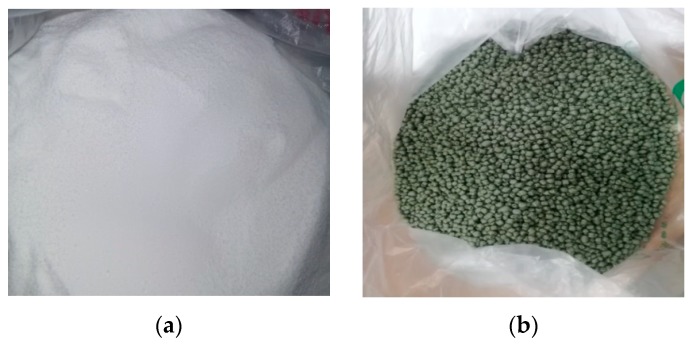
High modulus modifiers: (**a**) Honeywell7686; (**b**) Lubao.

The two high modulus modifiers (Figure 1), Honeywell 7686 (H7686) and Lubao, being highly efficient, convenient, and widely used in the production process, can be directly added to the aggregates.

Honeywell7686 (H7686), a white-powdered composite material with relatively low molecular weight, is soluble in asphalt. It is a special modifier and has the characteristics of rutting resistance, water resistance, and warm mixing construction. Lubao is a kind of high-density polyethylene material with good chemical stability and relatively high molecular quality, and is tasteless and non-toxic. The asphalt mixtures were reinforced by Lubao to improve its performance. The properties of modifiers H7686 and Lubao are shown in Table 3 and Table 4.

### 2.2. Aggregate Gradation

The type of the asphalt mix is AC-20 (dense gradation asphalt concrete-20). It belongs to hot mix asphalt mixtures, in which minerals of various particle sizes were designed according to the principle of dense gradation (marking in accordance with the Chinese standard of the Technical Specifications for Construction of Highway Asphalt Pavements (JTG F40—2004)). The continuous aggregate gradation, having a nominal particle maximum size of 19 mm, is listed in Table 5. According to the Marshall volumetric mix design, the 70-penetration and the SBS asphalts were directly mixed into the aggregates and the optimum asphalt binder content were 4.4% and 4.4% by weight, respectively. The mixing amounts of H7686 and Lubao were based on the best mixing amounts recommended by the manufacturer: 0.4% and 0.5% of the total mass of the asphalt mixtures, respectively. The two kinds of high modulus modifiers (Lubao and H7686) were directly added into the aggregates and the optimal ratios of binder were 4.6% and 4.5% by weight, respectively. The mixing temperature was 160°C, and the mixing time was 90 s. The asphalt mixtures with 70-penetration asphalt binder and the asphalt mixtures with SBS asphalt binder were respectively denoted Asphalt mix-70 penetration and Asphalt mix-SBS. And the Asphalt mix-70 penetration with the Lubao modifiers and the Asphalt mix-70 penetration with the H7686 modifiers were respectively denoted HMAM-Lubao and HMAM-H7686. The properties of the four kinds of asphalt mixtures are listed in Table 6.

### 2.3. Testing Results

**Figure 2:**
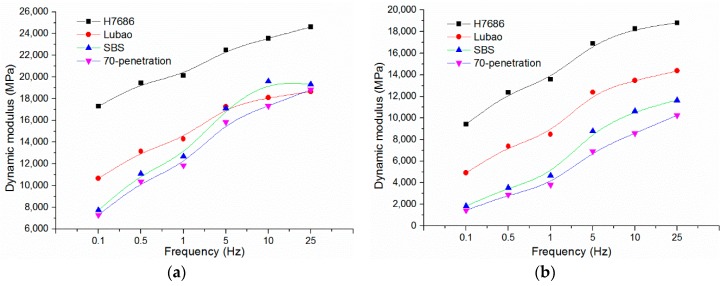

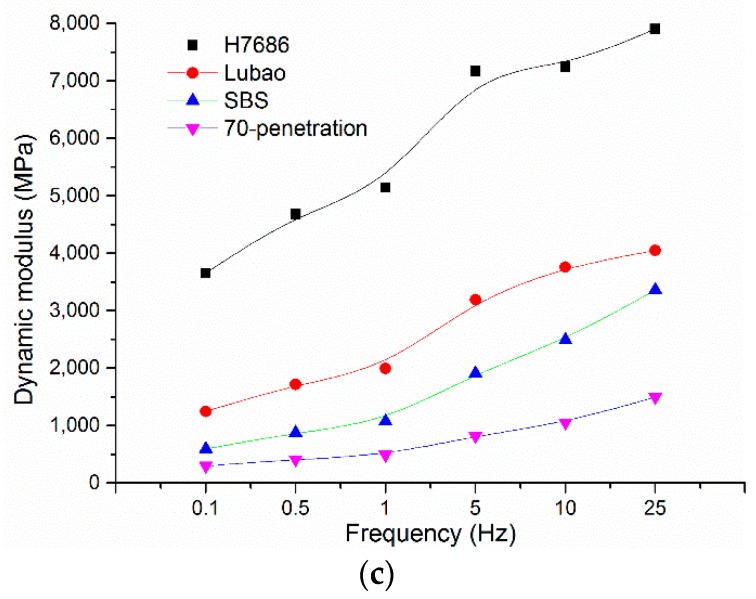
Dynamic modulus comparison results at different temperatures: (**a**) 5 °C; (**b**) 20 °C; (**c**) 45 °C.

A dynamic modulus test was conducted in accordance with the standard test methods of asphalt mixtures in China and the American Highway and Transportation Association standard AASHTO TP62-03. The Φ100 mm × 150 mm cylindrical test specimens were made by gyratory compaction, core drilling and cutting. Four kinds of specimens were tested for the dynamic moduli. Each specimen in this study was tested at 5, 20 and 45 °C, respectively, and the Haversine waveform was used as the loading method. At each test temperature, the load frequencies were 25, 10, 5, 1, 0.5, and 0.1 Hz, respectively, and a 60-s rest period was used between two neighboring frequencies. The dynamic test results of the four kinds of asphalt mixtures at the three temperatures and the six frequencies were automatically calculated by the microcomputer, as shown in Figure 2.

As shown in Figure 2, there are similar change laws between the HMAM and other asphalt mixtures changing with temperature and frequency in viscoelasticity. At a high temperature and a low frequency, the dynamic modulus of asphalt mixtures decreases, the elasticity is weakened, and the viscosity is enhanced; at low temperature and high frequency, it is the opposite. The dynamic modulus of asphalt mixtures can be significantly increased with the adding of a high modulus modifier. The dynamic moduli of the HMAM-H7686 are 1.4 and 1.3 times more than those of the Asphalt mix-70 penetration and the Asphalt mix-SBS at 5 °C and 25 Hz, respectively, and they are 5 times and 3 times at 45 °C and 25 Hz; The reason is that the high modulus modifiers can directly provide embedded packing, reinforcement, and cementation to and enhance the stiffness of the mixtures, and so the elastic features were enhanced.

### 2.4. Wheel Tracking Test

Rutting damage is one of the main diseases of the road surface. The laboratory investigations of the Asphalt mix-70 penetration, the Asphalt mix-SBS, the HMAM-Lubao and the HMAM-H7686 were carried out by wheel tracking test, in order to indicate a conventional performance related to the purpose of using the HMAM. The dynamic stability (*D_S_*), defined by Equation (1), and regarded as an indicator for directly characterizing the resistances to rutting of asphalt pavements, is positively correlated with rut resistance at high temperature. A rut board of 300 mm × 300 mm × 50 mm was made by a hydraulic sample-forming machine. The test was performed by the wheel tracking instrument at 60 °C. The resistance to deformations of the four kinds asphalt mixtures were measured based on the results of the wheel tracking test. The wheel tracking test results are listed in Table 7.

(1) $$D_{S}=\frac{\left(t_{2}-t_{1}\right)\times N}{d_{2}-d_{1}}$$

where *D_S_* is the dynamic stability, *t*_2_ is the point at 60 min, *t*_1_ is the point at 45 min, *N* is the speed, is usually 42 times·min^−1^, *d*_2_ is the deformation at *t*_2_ and *d*_1_ is the deformation at *t*_1_.

As shown in Table 7, the performance of the two HMAM at high temperature is obviously better than those of ordinary asphalt mixtures. The dynamic stabilities of the HMAM-H7686 and the HMAM-Lubao increase by 7 and 4 times, respectively, compared with the Asphalt mix-70 penetration. The high temperature performance of the HMAM-H7686 modifier is better than that of the HMAM-Lubao compared with the Asphalt mix-SBS, with increases of 2.5 and 1.5 times respectively.

## 3. Viscoelastic Parameters of High-Modulus Asphalt Mixture (HMAM)

Firstly, based on the results of dynamic modulus test, the master curves of the storage moduli were obtained by non-linear least-square fitting. The Wiechert mechanical model, which consists of 17 Maxwell models and a spring parallel, was then applied to describe its complex mechanical behavior. Finally, the time-domain Prony series expression of the relaxation modulus was obtained.

### 3.1. Master Curves of Storage Modulus

To carry out the conversion method for the Prony series, the master curves of the storage moduli were obtained, which was proposed by Park et al. [32,33,34]. The storage modulus was related to the dynamic modulus and the phase angle as [32]:

(2) $$E^{\prime }=\left|E^{*}\right|\cos\varphi$$

where *E*’ is the storage modulus, |E^*^| is the dynamic modulus and *φ* is the phase angle.

The sigmoidal function (Equation (3)) was selected to describe the master curves of the storage moduli [32]:

(3) $$\log\left|E^{\prime }\right|=\delta +\frac{Max-\delta }{1+e^{\beta +\gamma \log\omega_{r}}}$$

where *ω_r_* is the reduce frequency, *Max* and *δ* are as lg logarithmic form for the maximum and minimum values of the dynamic modulus, respectively, *β* and *γ* are the shape parameters related to the properties of the mixtures. Parameter *γ* influences the steepness of the function (rate of change between the minimum and the maximum) and *β* is the horizontal position of the turning point.

The Arrhenius equation was used to calculate the reduced frequency ω*_r_* at reference temperature 20° C [35] and was defined as:

(4) $$\log\omega_{r}=\log\omega +\frac{\Delta E_{a}}{19.14714}\left(\frac{1}{T}-\frac{1}{T_{r}}\right)$$

where *ω* is the frequency at the reference temperature, Δ*E_a_* is the activation energy, *T* is the test temperature and *T_r_* is the reference temperature.

The data of the storage modulus were fitted by non-linear least-squares method and the parameters *δ* and *β* in Sigmoidal function were obtained by using the programming solving function in Excel, as shown in Table 8 [36]. The shift factor *α*(*T*) [37] was obtained based on the principle of time-temperature equivalence using Equation (5) [25], i.e.,

(5) $$\log\left[\alpha (T)\right]=\frac{\Delta E_{a}}{19.14714}\left(\frac{1}{T}-\frac{1}{T_{r}}\right)$$

**Figure 3:**
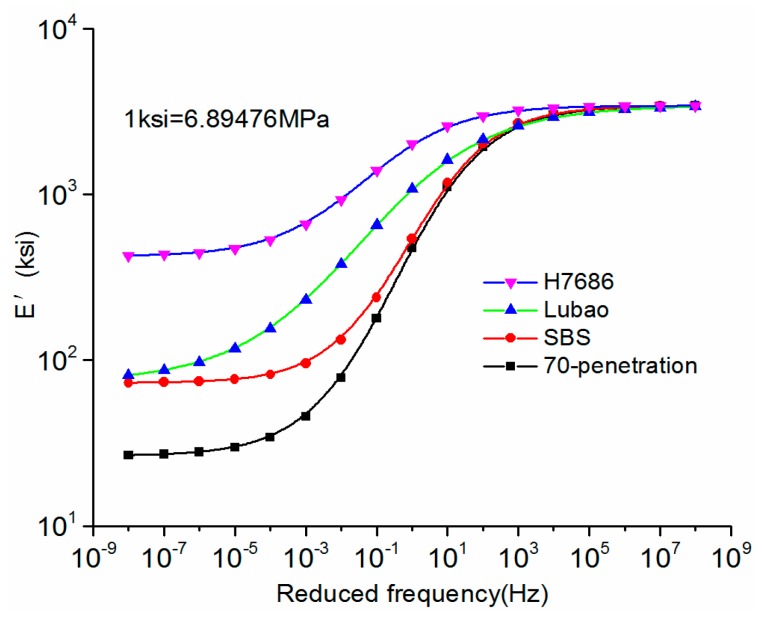
Master curves of the storage moduli at reference temperature 20 °C.

Based on the parameters in Table 8, the master curves of the storage moduli of four kinds of asphalt mixtures at the reference temperature were obtained. The master curves of the storage moduli at 20 °C are shown in Figure 3. In addition, the sigmoidal function equations of other main temperature curves could be obtained and the master curves could be drawn basing on the same non-linear fittings.

As shown in Figure 3, at low frequencies, the storage moduli of the HMAM were much higher than those of other asphalt mixtures. The loading frequency actually corresponded to the vehicle speed, and the speed decreased as the frequency was reduced. Therefore, the adverse effect with a low speed could effectively be resisted by the HMAP. In addition, the changing rates of the storage moduli of the HMAM were slower, which means that the HMAP was insensitive to the variation of the speeds of the driving load.

### 3.2. Maxwell Model Parameters

According to theoretical and experimental research, more complex and multivariate models were needed to accurately represent the long-term complex viscoelastic behaviors of asphalt mixtures [38,39]. The Maxwell model with the Wiechert mechanical model is a common mechanical analysis model, which is composed of several Maxwell models and a spring in parallel and can be used to describe more complex mechanical behaviors [40]. In this paper, the Wiechert mechanical model was used to fit the data. The correlation coefficient was greater than 0.99, which showed good agreement. The Wiechert model could also be used to obtain time-domain Prony series expression of the relaxation modulus [41]. The relaxation modulus *E*(*t*) is written as [32]:

(6) $$E\left(t\right)=E_{\infty }+\sum_{m=1}^{M}E_{m}\exp\left(-t/\rho_{m}\right)$$

where *m* is the number of parallel models, *E*_∞_ is the infinite relaxation modulus, *E_m_* is the relaxation modulus in the *m*_th_ term or Prony coefficient and *ρ_m_* is the relaxation time.

The total stress in the Wiechert model was obtained by the summation as [39]:

(7) $$\sigma_{m}=\sigma_{\infty }+\sum_{m=1}^{M}\sigma_{m}$$

where *σ* is the total stress, and *σ*_∞_ is the limit stress when angular frequency *ω* approaches 0 from the right side.

The stress, *σ_m_*, in each of the Maxwell components combining a spring with a dashpot is governed by the differential equation [39]:

(8) $$\frac{d\varepsilon }{dt}=\frac{1}{E_{m}}\frac{d\sigma_{m}}{dt}+\frac{\sigma_{m}}{\eta_{m}}$$

where *η_m_* is the coefficient of viscosity, *E_m_* is the relaxation modulus in the *m*_th_ term or Prony coefficient, and *ε* is the strain. The number of terms *m* used in the fitting is equal to the number of decades for which the fitting is to be done.

Due to the linearity of the material components, the total stress in the Wiechert model is obtained by Equation (9) [39]:

(9) $$\sigma_{\infty }=E_{\infty }\varepsilon$$

where *σ*_∞_ is the stress in the *m*_th_ term, *E*_∞_ is the limit storage modulus when angular frequency *ω* approaches 0 from the right side and *ε* is the strain.

By using the relaxation time expression *ρ_m_* = *η_m_*/*E_m_*, the time-domain could be converted into the frequency-domain. The Prony series expression of the storage modulus can be obtained from Equation (10) as [32]:

(10) $$E^{\prime }\left(\omega \right)=E_{\infty }+\sum_{m=1}^{M}\frac{\omega^{2}\rho_{m}^{2}E_{m}}{\omega^{2}\rho_{m}^{2}+1},\;m=1,2,\ldots M$$

The mechanical parameters of the Prony series of the relaxation modulus could be fitted according to the master curves of the storage modulus after determining the relationship between the storage modulus and the Prony series of the relaxation modulus [42,43]. To solve *E_m_* and *ρ_m_*, the collocation method was usually used rather than solving a nonlinear system of equations with 2*m* unknowns because of the 10^−8^–10^8^ frequency range of the dynamic modulus master curves. A series of relaxation time points were set in advance, and then the parameters corresponding to these relaxation time points could be solved [29]. According to the research, if the distance between relaxation time points was too small, more points needed to be taken, and if the distance was too large, there would be a large fluctuation about the relaxation modulus curve. The relaxation modulus curve determined was stable and there was no fluctuation when the distance between relaxation time points was about 1 on the log(*ρ_m_*) axis [33,34]. Therefore, when the distance on the log(*ρ_m_*) axis was taken as unit 1, 17 parallel Maxwell models, namely 17 value groups of *E_m_*s and *ρ_m_*s, would be generated. The relaxation time points of *ρ*_1_–*ρ*_17_ were determined first, and then the fitting was carried out. In this way the calculations were simplified and the accuracy of the calculated relaxation curve was ensured. The relaxation time points were usually determined in the following form [32]:

(11) $$\rho_{m}=2\times 10^{\left(m-c\right)},\;m=1,2,\ldots 17$$

where *c* is determined according to the range of test specimens and the purpose of the research, in the case of asphalt mixtures, more than 10 relaxation time points should usually be preconfigured.

The fitting could be carried out according to the master curves of the storage moduli after the parameters were determined. In this paper, the parameters of the Prony series expression at 20 °C were obtained, as shown in Table 9. The viscoelastic variation laws of different asphalt mixtures were obtained, and the mechanical response analysis of asphalt mixtures based on actual parameters could be carried out.

## 4. Calculation of Mechanical Responses of Viscoelastic HMAP under Moving Load

### 4.1. 3D Viscoelastic Finite Element Model (FEM) of Pavement under Moving Load

In this paper, the models of asphalt pavements for the viscoelastic structures were built using ABAQUS, and *E_m_* can be interconverted from the expression of Prony series of the relaxation modulus. *E_m_* was transformed into *g_i_* (the ratio of each elastic modulus to the sum) based on the set requirements of ABAQUS. The model had a dimension of 6 m along the direction of traffic, 6 m across the transverse direction, and 6 m in depth. A 3D model of the same size was built to minimise the edge effect and achieve one full passage of the truck on the pavement to obtain a complete longitudinal strain and stress response curve including the expected compression–tension–compression sequence [24]. There are many advantages in using a 3D FEM: first, the 3D FEM allows the consideration of complex behaviors of pavement material; second, it allows the simulation of different complex situations; third, the analysis results may substitute for the tests. However, the simulation process of moving loads and dynamic analysis require a huge amount of computation [24]. In our model, the *x*-axis was perpendicular to the wheel path (transverse), the *y*-axis was along the wheel path (longitudinal), and the *z*-axis was vertical. To improve the rate of convergence, eight-node brick elements with reduced integration (C3D8R) were used and the pavement model consisted of 66,650 elements and 72,072 nodes. Full interface bonding was assumed between all layers, and the bottom boundary of the model was in full constraint, the side boundary was constrained in the normal direction. The traditional loading method is static loading, which is not in accordance with the actual pavement stress, so it is important to carry out the dynamic analysis of asphalt pavement subjected to traffic load [44]. In the study, the dynamic responses caused by moving load on the HMAP were considered. Therefore, to simulate a moving load, the tyre–pavement contact area was progressively shifted along the wheel path in the direction of traffic until a single tyre pass is completed [45]. The contact area of a truck tyre is in reality closer to a rectangular than to a circular shape regardless of the types of tyre [46]. For the application of moving load, the two wheel moving paths were set symmetrically along the direction of wheel moving load and were refined, which had a dimension of 4 m along the direction of traffic and 0.186 m across the transverse direction. The center distance between the two wheel moving paths was 0.314 m, which was in accordance with the standard truck of China. The wheel path had a length of 4 m along the direction of traffic, and a driving distance of 0.2s at a speed of 72 km/h. The wheel path was discretized in time and space to apply the Haversine wave load, and the Haversine function that changes over time was applied to the wheel moving path.The Haversine function was written as:

(12) $$Q(t)=p_{\max}\sin^{2}\left(\frac{\pi }{2}+\frac{t}{d}\right)$$

where *d* is the duration of load that depends on the speed *v* and the wheel contact area radius *a*. It is generally believed that when the load is 6*a* away from a point, the load has no effect on the point, so we have *d* = 12*a*/*v*. When the load is far from the known point, or *t* = ±*d*/2, *Q*(*t*) = 0. When the load directly acts on the point (*t* = 0), the load reaches the peak value, and the load pressure is *p*_max_. The simulation of the driving load has been shown in reference [47].

**Figure 4:**
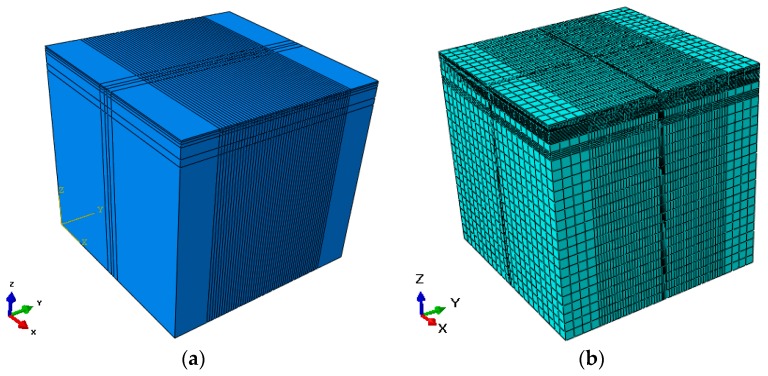
3D pavement finite element model (FEM): (**a**) FEM; (**b**) mesh part.

The analyses of stresses under standard load (*p_max_* = 0.7 MPa) and heavy load (*p_max_* = 1.0 MPa) were conducted. In the analysis, the meshes of the loading area were refined. The shearing stresses at depths from 0.04 m to 0.10 m of the asphalt pavement were the main focuses, and the resistance to rutting deformation was mainly provided by the middle surface layer, so the HMAM was set in the middle surface layer. Four kinds of asphalt pavements (the Asphalt mix-70 penetration, the Asphalt mix-SBS, the HMAM-Lubao, the HMAM-H7686) were used for the middle surface layer to analyze the mechanical responses, and their stress characteristics and change laws were studied. The FEM model is shown in Figure 4 and the selection of pavement structure parameters were referred to the Specifications for Design of Highway Asphalt Pavement (JTG D50-2017), as shown in Table 10. The viscoelastic parameters of upper and under layer materials come from reference [48].

### 4.2. Mechanical Responses of Pavement Structures

**Figure 5:**
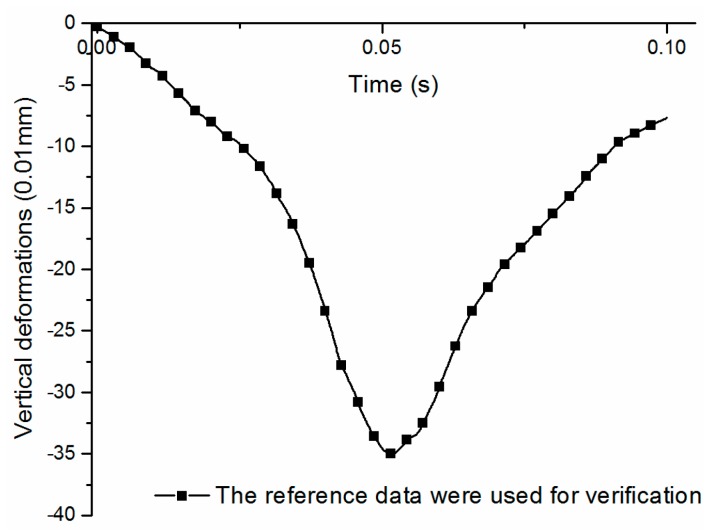
Curves obtained by literature data.

To verify the accuracy of the model established in this paper, the results were compared with those in reference [48]. The parameters of the pavement structures were from reference [48]. The settings of the model size, the drive speed, the drive distance and the Haversine wave load were the same as in the reference. The vertical deformations time-history curve of the road surface was obtained by using the model built in this paper, as shown in Figure 5.

**Figure 6:**
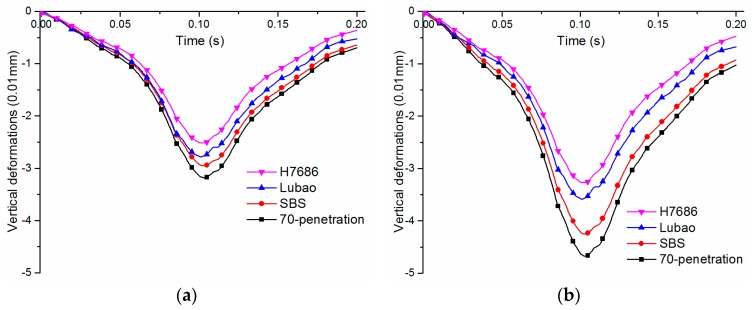
Vertical deformations at centers of road surface: (**a**) under standard load; (**b**) under heavy load.

Comparing Figure 6 in this paper with Figure 5 in reference [48], it can be seen that the trend of two curves was same, the peak value of the deformations only differed by 0.19 mm, and the error is within the allowable range of finite element analysis, which verifies the correctness and feasibility of the numerical simulations in this paper.

#### 4.2.1. Vertical Deformations at Road Surface

The damage of the road can be caused by the surface deformation in the process of vehicle driving, and the safety factor would reduce. In this paper, the time-varying vertical deformations at the center of different pavements surface under *p_max_* = 0.7 MPa and 1.0 MPa were considered. As shown in Figure 6, there is a typical asymmetric distribution on both sides of the curve because of the viscoelasticity of asphalt mixtures. The two kinds of HMAP have more remarkable resistances to deformations under different load levels. Compared with the 70-penetration asphalt pavement, the deformation of the HMAP under 0.7 MPa reduces by about 20%–25%; the resistances to deformations of the HMAP are more prominent under heavy load, with a decline from 29% to 36%. The load levels have a great influence on the vertical deformations on the pavement surface. The vertical deformations at the pavement surface increase significantly with the load levels. The vertical deformation of the Asphalt mix-70 penetration pavement increases by about 50%, the Asphalt mix-SBS pavement increases by 45%, and the HMAP-Lubao pavement and the HMAP-H7686 pavement increase by 29 and 31%, respectively, under the heavy load. The use of the HMAM in the pavement reduces the road damages and the sensitivity to traffic loads, and at the same time increases the rutting resistance and durability.

#### 4.2.2. Shearing Strains of Middle Surface Layers

**Figure 7:**
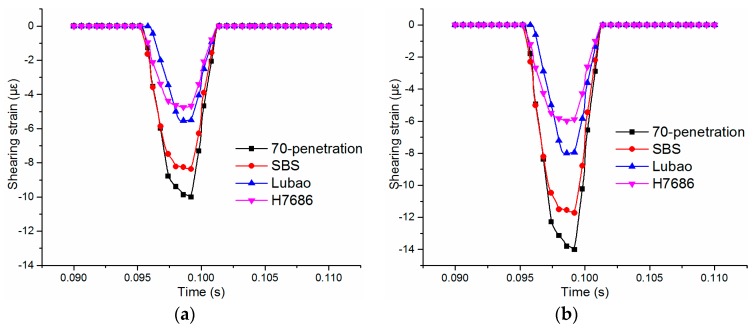
Shearing strains of middle surface layers: (**a**) under standard load; (**b**) under heavy load.

The rutting problem of asphalt pavements was mainly caused by the shearing deformations of the asphalt layer and the compaction failure of vehicle reciprocating. The shearing strains of the asphalt pavement were mainly concentrated at depths from 0.04 m to 0.10 m, located in the middle surface layer [19] and, therefore, it is necessary to analyze the shearing strains of the middle surface layer. The time-history curves of the shearing strains are shown in Figure 7. It can be seen that the modulus of the HMAP has greater influence on the maximum shearing strain of the pavement structures. The peak values of the shearing strain of the middle surface layer decrease significantly with the increasing of the moduli. Compared with the Asphalt mix-70 penetration pavement, the shearing strain of the HMAP-H7686 pavement can be reduced by as much as 8 με, and the decreasing rate was 57%. The peak values of the shearing strains of the middle layer increase to different degrees with the load levels, and the similar change laws of the shearing strains under standard and heavy loads were obtained.

#### 4.2.3. Stresses and Strains at Bottom of Underlayers

**Figure 8:**
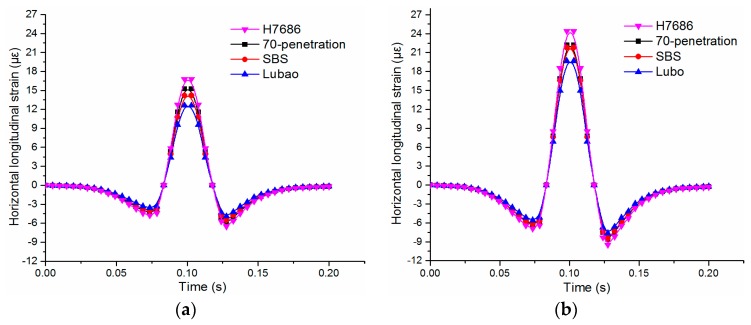
Horizontal longitudinal tensile strains at the bottom center of underlayers: (**a**) under standard load; (**b**) under heavy load.

The transverse strain of the asphalt layer under the symmetrical load was 0 since the plane of the vehicle moving along the wheel path was a symmetrical plane, so the transverse strain analysis of the bottom was not performed. The time-history curves of the tensile strain at the bottom center of the underlayer under the two load levels are shown in Figure 8. As shown in Figure 8, the change laws are similar for both situations. The alternating change of pressure-pull-pressure occurs at the bottom of the asphalt layer after loading, which was consistent with the viewpoints in reference [28,29].

The fatigue damages of asphalt pavements were easily caused by extortionate tensile stress and tensile strain. The HMAM-H7686 pavement has the highest tensile strain, followed by the Asphalt mix-70 penetration pavement, the Asphalt mix-SBS pavement and the HMAM-Lubao pavement. The peak values of the tensile strains increase significantly with the loads, and the four kinds of asphalt mixtures increase by 40% to 52%. Although the dynamic modulus of the HMAM-H7686 was larger than that of the HMAM-Lubao, the tensile strains at the bottom of the underlayer cannot be effectively decreased by the HMAM-H7686 pavement, and the tensile strain of the HMAM-Lubao asphalt pavement reduces by 16% compared with that of the HMAM-H7686 pavement.

**Figure 9:**
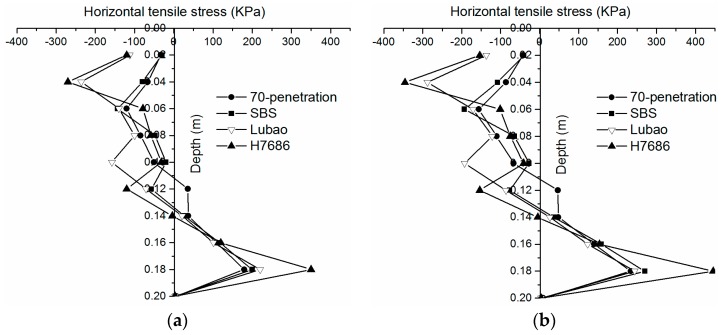
Horizontal longitudinal tensile stresses at the bottom center of underlayers: (**a**) under standard load; (**b**) under heavy load.

The tensile stress and strain need to be considered together, for the cracking occurred at the bottom of the asphalt layer. The cracking may be associated with tensile stress at the bottom of the road surface. The tensile stresses at the bottom layer in different depths were extracted downward along the center of the wheel gap in order to explore the reason of cracking. As shown in Figure 9, there is obvious excessive tensile stress at the bottom of the underlayer of the HMAM-H7686 pavement. The tensile stresses are about 70% and 110% higher than those of the HMAM-Lubao and the Asphalt mix-70 penetration pavements, respectively. The excessive tensile stress at the bottom of the layer was produced after setting of the HMAM-H7686 layer, resulting in a larger peak value of the tensile strain. The excessive tensile stress at the bottom of the asphalt layer may be due to the too-large modulus, and the risk of cracking of the HMAM is significantly increased; therefore, it is necessary to effectively control the ranges of the moduli under the premise that the resistance to deformations of the structure should be satisfied in the selection of materials.

**Figure 10:**
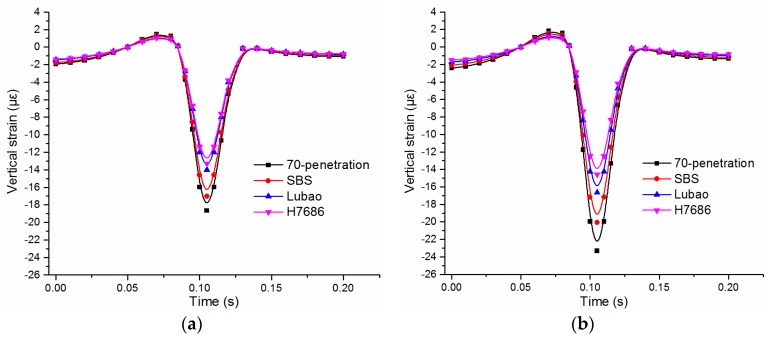
Vertical strains at bottom center of underlayers: (**a**) under standard load; (**b**) under heavy load.

The time-history curves of the vertical strain at the bottom of the underlayers under standard and heavy loads are shown in Figure 10, and similar change laws are observed in both cases. After loading, the alternating change of pull-pressure occurs at the bottom of the asphalt layer. A typical asymmetrical distribution appears on both sides of the curve due to the viscoelasticity of asphalt mixtures. The pavements sorting by the peak values of the vertical strains in descending order are: the Asphalt mix-70 penetration pavement, the Asphalt mix-SBS pavement, the HMAM-Lubao pavement and the HMAM-H7686 pavement. HMAP show good resistances to vertical deformations. As the pavement structure with higher modulus, the vertical strains of the HMAM-H7686 asphalt pavement reduce by 6% and 14% under standard and heavy loads compared with the HMAM-Lubao pavement. It can be seen that the resistance of the asphalt pavement to vertical deformations at the bottom of the layers is significantly enhanced due to the increasing dynamic modulus.

#### 4.2.4. Compressive Strains at Top Center of Subgrades

**Figure 11:**
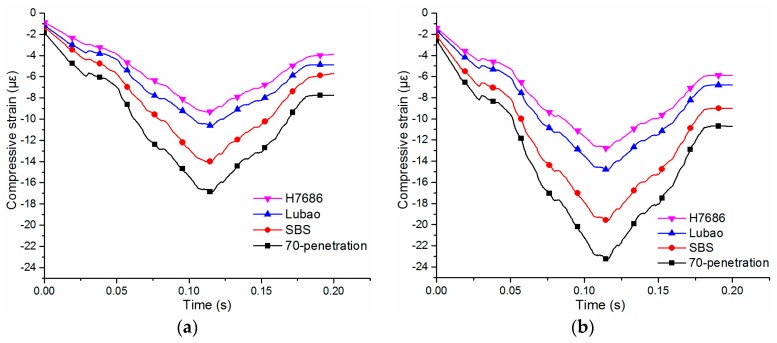
Compressive strains at top center of subgrades: (**a**) under standard load; (**b**) under heavy load.

The resistance to overall deformations of pavements can be reflected by the compressive strain at the top center of the subgrade. The time-history curves of the compression strains at the top center of the subgrade under standard and heavy loads are shown in Figure 11. The compressive strain begin to appear and gradually increase to the peak value when the wheel is near the center, and the time-history curves are asymmetric. The pavements sorting by the peak values of the compressive strains at the top center of the subgrade in descending order are: the Asphalt mix-70 penetration pavement, the Asphalt mix-SBS pavement, the HMAM-Lubao pavement and the HMAM-H7686 pavement. The compressive strains at the top surface of the HMAM-H7686 pavement and the HMAM-Lubao pavement decrease by 40% and 42%, 35% and 26%, respectively, compared with those of the Asphalt mix-70 penetration pavement under standard and heavy loads. Above all, similar to the change laws of the vertical strain of the underlayer, there is a remarkable resistance to vertical deformations in the HMAP.

### 4.3. Sensitivity of HMAP to Level of Loads

In order to analyze the effect of increasing load levels on the mechanical responses of different asphalt pavements, the peak values of the vertical deformations at the center of the road surface, the shearing strains of the middle surface layer, the vertical strains at the bottom center of the underlayer, and the compressive strains at the top center of the subgrade were extracted. The increasing rates of the mechanical responses are obtained with the load change, as shown in Table 11.

As shown in Table 11, the HMAP can not only improve the resistance to deformation, but also reduce sensitivity to the variation of the traffic loads, and at the same time enhance the rutting resistance and durability. However, the increasing rates of the tensile strain at the bottom of asphalt layer of the HMAP are similar to those of other pavements, reaching more than 42%, illustrating that the stress concentration at the bottom of the asphalt layer is more serious as the loads increase, and cracking at the bottom of the asphalt layer is more prone to occur. Therefore, it was necessary to control the tension at the bottom of the asphalt layer effectively under the premise that resistance to the deformations of the structure should be satisfied in practice.

## 5. Conclusions

A more extensive characterization of the mixtures was presented by a wheel tracking test. The dynamic moduli were tested by laboratory investigations and the viscoelastic parameters were obtained. The 3D viscoelastic FEM of pavements under moving loads were established and the mechanical responses were analyzed; the mechanical characteristics of the pavement structures after the setting of the high-modulus layer and the sensitivity of various pavement structures to the level of the loads were objectively analyzed to provide the theoretical basis for improving the structure design of the HMAP, and the following conclusions can be drawn:

(1) The wheel tracking test results indicate that the addition of the high-modulus modifiers can improve the high-temperature stability of the pavement. Dynamic modulus tests were successfully conducted to obtain the viscoelastic parameters and study the mechanical properties of the HMAM. The results will be helpful in interpreting the modifier behavior by explaining the change laws in the viscoelastic parameters with the loading temperature and frequency.
(2) The changing rates of the storage modulus curves of the HMAM were slower than those of other asphalt mixtures, which mean that the HMAP were insensitive to the variation of the speeds of the driving load.
(3) The impacts of the viscoelasticity on the mechanical responses of different pavements were identified, and the process of the mechanical responses was represented by the suggested model. The vertical deformations of the road surface, the shearing strains of the middle surface layer, the vertical strains of the underlayer, and the compressive strains of the subgrade of the two HMAP were significantly lower than those of two other pavements, and the two HMAPs perform well against the deformations. The alternating change of pull-pressure occurs at the bottom of the asphalt layer after loading and a typical asymmetric distribution appears on both sides of the curves due to the viscoelasticity of the asphalt mixtures.
(4) The tensile strain and stress at the bottom of the underlayer of the HMAM-H7686 pavement do not decrease with the increase of the dynamic modulus, illustrating that the change laws of the tensile strain and stress are affected by the range of the dynamic modulus. The HMAM-H7686 pavements have the highest tensile strain, followed by the Asphalt mix-70 penetration pavements, the Asphalt mix-SBS pavements and the HMAM-Lubao pavement. The tensile strains at the bottom of the under layer cannot be effectively reduced by the HMAM-H7686, which has the highest dynamic modulus. There were obvious excessive tensile stress at the bottom of the underlayer for the HMAM-H7686 pavement, and the tensile stresses was about 70%, 110% higher than those of the HMAM-Lubao pavements and the Asphalt mix-70 penetration pavements, respectively. In conclusion, the range of the modulus of the materials must be controlled to avoid cracking at the bottom of the layer when the HMAM is selected.
(5) The load levels have great effects on the mechanical responses, and the degrees to which the mechanical response is affected by different load levels were discussed in detail. The HMAP is insensitive to the load changes and could better withstand the adverse effects of the heavy load. But the increasing rates of more than 42% of the tensile strain at the bottom of the underlayer of the HMAP were similar to other pavements, which means that the tensile stress at the bottom of the asphalt layer is more serious as the loads increase, and the bottom of the asphalt layer is more prone to cracking.

## Figures and Tables

**Table 1:** Properties of 70-Penetration asphalt.

Properties	Unit	Value	Method
Penetration (25 °C, 100 g, 5 s)	0.1 mm	63.2	T0604-2011
Softening Point	°C	49.1	T0606-2011
Ductility (5 cm/min, 15 °C)	cm	>100	T0605-2011
Viscosity (177 °C)	Pa·s	3.8	T0625-2011

**Table 2:** Properties of styrene-butadiene-styrene (SBS) asphalt.

Properties	Unit	Value	Method
Penetration (25 °C, 100 g, 5 s)	0.1 mm	50.7	T0604-2011
Softening Point	°C	75.6	T0606-2011
Ductility (5 cm/min, 15 °C)	cm	>200	T0605-2011
Viscosity (177 °C)	Pa·s	4.1	T0625-2011

**Table 3:** Properties of H7686.

Properties	Unit	Value
Droplet Point	°C	130–138
Viscosity—150 °C Brookfield	Pa·s	4.1–4.8

**Table 4:** Properties of Lubao.

Properties	Value	Standards
Tensile Strength/MPa	18.8	18–20
Elongation at Break/%	102.8	≥100
Density/(g·cm^−3^)	0.94	0.93–0.96
Melt Flow Rate/(g/10min)	1.2	1–4
Vicat Softening Temperature/°C	61.7	≤140
Resin Content/%	98.89	≥95
Particle Diameter/mm	3.7	3–5

**Table 5:** Aggregate gradation.

Sieve Size/mm	Upper Limit/%	Lower Limit/%	Gradation/%
26.5	100	100	100
19	100	90	93.7
16	92	78	86.3
13.2	80	62	79.2
9.5	72	50	66.3
4.75	56	26	45.9
2.36	44	16	28.1
1.18	33	12	20.0
0.6	24	8	13.2
0.3	17	5	8.1
0.15	13	4	5.4
0.075	7	3	3.6

**Table 6:** The properties of asphalt mixtures.

Materials	Asphalt Contents/%	Relative Bulk Volume Density/g·cm^−3^	Air Voids/%	Stability/kN	Flow Value/mm
Asphalt mix-70 Penetration	4.4	2.422	4.1	14.2	3.07
HMAM-Lubao	4.6	2.417	3.8	22.5	3.77
HMAM-H7686	4.5	2.420	3.9	21.32	3.69
Asphalt mix-SBS	4.4	2.411	3.8	19.38	3.46

**Table 7:** The wheel tracking testing results.

Materials	Rut Deformation at 45 min (mm)	Rut Deformation at 60 min (mm)	DS (times·mm^−1^)
Asphalt mix-70 Penetration	3.781	4.182	1571
Asphalt mix-SBS	2.160	2.303	4405
HMAM-Lubao	1.403	1.501	6428
HMAM-H7686	0.680	0.739	10,857

**Table 8:** Main parameters and shift factors of the master curves at reference temperature 20 °C.

Materials	*δ*	*β*	*γ*	Δ*E_a_*	lg (Shift Factors)		
					5 °C	20 °C	45 °C
Asphalt mix-70 penetration	1.4229	−0.37382	−0.81492	169,829	1.6311	0	−2.3768
Asphalt mix-SBS	1.8628	−0.07669	−0.88450	165,627	1.5907	0	−2.3179
HMAM-Lubao	1.8669	−0.83931	−0.56673	208,895	2.0063	0	−2.9235
HMAM-H7686	2.5891	−1.04341	−0.79735	186,848	1.7946	0	−2.6149

**Table 9:** Parameters in Prony series representations for relaxation moduli at 20 °C.

Relaxation Time Points	*ρ_m_*/s	Asphalt Mix-70 Penetration	Asphalt Mix-SBS	HMAM-Lubao	HMAM-H7686
		*E*_m_/MPa			
1	2.0 × 10^−8^	124.69	79.43	338.30	32.47
2	2.0 × 10^−7^	195.62	137.46	380.33	50.11
3	2.0 × 10^−6^	446.27	336.56	673.41	113.59
4	2.0 × 10^−5^	966.78	787.18	1111.94	247.91
5	2.0 × 10^−4^	2008.84	1769.92	1776.08	535.40
6	2.0 × 10^−3^	3759.50	3581.76	2637.22	1116.83
7	2.0 × 10^−2^	5664.62	5742.67	3496.84	2167.92
8	2.0 × 10^−1^	5629.70	5783.80	3904.68	3627.44
9	2.0 × 10^0^	3124.47	3134.60	3483.50	4630.18
10	2.0 × 10^1^	1050.41	1086.52	2409.40	3982.63
11	2.0 × 10^2^	335.83	381.05	1407.16	2417.14
12	2.0 × 10^3^	104.25	125.17	616.44	1041.63
13	2.0 × 10^4^	65.22	78.35	568.91	803.59
14	2.0 × 10^5^	42.22	53.33	128.24	105.46
15	2.0 × 10^6^	0.00	38.56	2.33	0.76
16	2.0 × 10^7^	18.11	20.62	208.97	244.21
17	2.0 × 10^8^	25.56	2.13	24.38	295.42
*E*_∞_/MPa		182.5676	502.7753	507.55	2077.00

**Table 10:** Parameters for pavement structure layers.

Structure Layers	Thickness/mm	Mechanical Parameters/MPa	Poisson’s Ratio
Upper layer (SMA-13)	40	viscoelasticity	0.25
Middle surface layer (Four kinds of materials) (AC-20)	60	viscoelasticity	0.25
Underlayer (AC-25)	80	viscoelasticity	0.25
Base	300	7500	0.25
Subbase	200	250	0.35
Subgrade	/	100	0.4

**Table 11:** Increasing of mechanical responses from standard to heavy loads.

Sample Types	Asphalt Mix-70 Penetration	Asphalt Mix-SBS	HMAM-Lubao	HMAM-H7686
	Increasing Rates of Mechanical Responses/%			
Vertical deformations of the road surface	47	44	29	30
Shearing strains of the middle surface layer	40	45	37	25
Horizontal tensile strains of asphalt layer bottom	49	52	42	45
Vertical strains of asphalt layer bottom	24	17	18	9
Compressive strains at the top of the subgrade	42	39	36	32
